# Liposomes as Multifunctional Nano-Carriers for Medicinal Natural Products

**DOI:** 10.3389/fchem.2022.963004

**Published:** 2022-08-08

**Authors:** Xiamin Cheng, Hui Yan, Songhao Pang, Mingjun Ya, Feng Qiu, Pinzhu Qin, Chao Zeng, Yongna Lu

**Affiliations:** ^1^ Institute of Advanced Synthesis, School of Chemistry and Molecular Engineering, Jiangsu National Synergetic Innovation Center for Advanced Materials, Nanjing Tech University (Nanjing Tech), Nanjing, China; ^2^ School of Environment and Ecology, Jiangsu Open University, Nanjing, China; ^3^ The Third Affiliated Hospital of Zhejiang Chinese Medical University, Hangzhou, China

**Keywords:** liposome, nano-carrier, targeted-delivery, natural product, traditional Chinese medicine

## Abstract

Although medicinal natural products and their derivatives have shown promising effects in disease therapies, they usually suffer the drawbacks in low solubility and stability in the physiological environment, low delivery efficiency, side effects due to multi-targeting, and low site-specific distribution in the lesion. In this review, targeted delivery was well-guided by liposomal formulation in the aspects of preparation of functional liposomes, liposomal medicinal natural products, combined therapies, and image-guided therapy. This review is believed to provide useful guidance to enhance the targeted therapy of medicinal natural products and their derivatives.

## Introduction

Traditional Chinese medicine (TCM) and other herbal medicines have been used in various therapies for thousands of years (Cook et al., 2013). Especially, TCM has been accepted widely in East Asian countries (China, Korea, and Japan) and Southeast Asia countries because of many promising effects against diseases including cancer, infections, murrain, and so on. The key is a wide spectrum of bioactive natural products in the fruit, leaf, flower, root, stem, rhizome, and bark parts of medicinal plants, bacteria, and even special parts of animals, such as alkaloids, polysaccharides and terpenoids, exhibiting anti-inflammation, anti-tumor, anti-hepatic fibrosis, immunosuppression and other effects (Atanasov et al., 2021; Shao et al., 2021). Therefore, natural product has become one of the most important sources of drug discovery. However, they often suffered drawbacks that restricted their applications in disease treatment. First of all, the low solubility and stability in physicochemical environments (blood, body fluid, and low pH in digestion) and rapid blood clearance whereas it is compulsory to retain efficient drug concentration in the therapy. For those hydrophilic natural products, although the solubility is good, they were probably secreted out by the quick renal clearance (Varma et al., 2009). Second, the side effects, i.e., the potential toxicity to unwanted sites, and low bio-availability, occur when the delivery and treatment lack targeting due to multiple biological barriers, such as blood–brain barrier or blood–tumor barrier (Blanco et al., 2015). Thirdly, for the treatments, such as *anti*-cancer and *anti*-microorganism, drug-resistance to a single therapy often besets pharmacists. Finally, the modification of the molecule of natural product to solve the problems is a relatively more difficult, cost- and time-consuming task. Therefore, in clinical, suitable drug formulation emerges as an alternative solution.

As one of the first drug delivery systems, liposome formulation has been used universally in clinical trials and clinics (Gregoriadis, 1976). The nanoparticles consisting of natural/synthetic lipid, drug, additives, and surface modification moiety, have shown excellent advantages in drug delivery: *1*) The physical compartment of the lipid bilayer protects the natural product and other cargos from erosion under physiological environments. It also reduces the risk of drug exposure to non-lesion sites. *2*) The nano-size, good stability, and multifunctions enhance the targeted delivery and controllable drug release (Jain and Jain, 2018). The enhanced permeability and retention (EPR) effect enhances the tumor-targeting delivery of nanoliposome. Furthermore, the lipid membrane promotes cell uptake by endocytosis. *3*) The big capacity for hydrophobic and hydrophilic cargoes (e.g. drug and contrast agents) is suitable for combined therapies and image-guided therapy to enhance the therapeutic effects, reduce side effects, and drug resistance. *4*) The natural lipid has good biocompatibility.

In past decades, liposome has exhibited an excellent nano-platform for natural products in drug delivery. A comprehensive review is compulsory to summarize the achievements and reflect on the problems as useful guidance for future development.

## Construction of Liposomal Systems for Medicinal Natural Products

### Structure and Preparation Methods

**FIGURE 1:**
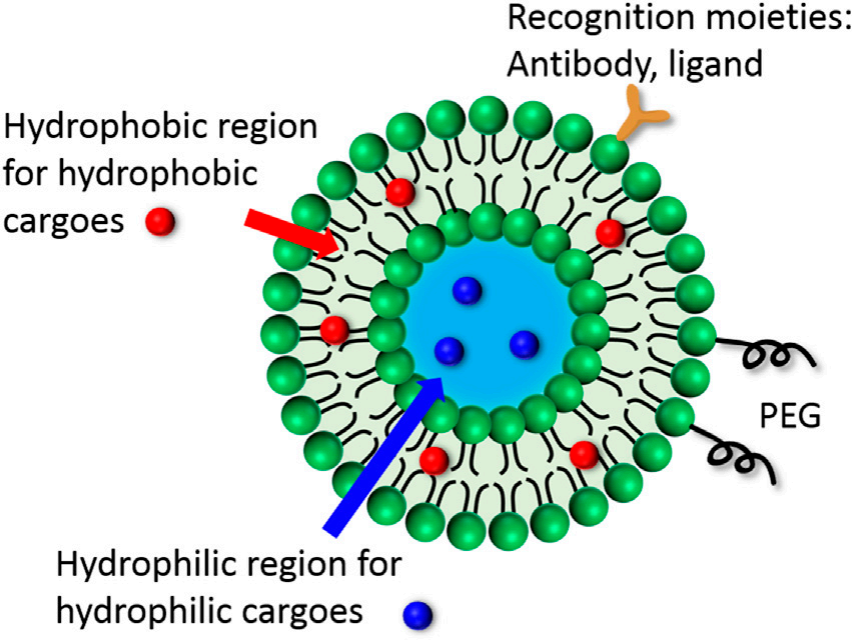
Typical structure of liposome.

In liposome, an aqueous core is entrapped by a lipid bilayer (Figure 1). Therefore, the liposome becomes an ideal carrier for hydrophobic (aqueous core) and hydrophilic (lipid bilayer) payloads, respectively. To resist the self-aggregation and body clearance mechanism, PEG modification on the surface has become a routine strategy. To realize the targeted delivery and therapy, functional modifications have been introduced to the liposome surface or embedded in the bilayer for active-targeted delivery and triggerable release.

The liposome is constructed by several strategies such as thin-film hydration, ethanol injection method (Jaafar-Maalej et al., 2010), active drug encapsulation (Gubernator, 2011). Besides typical liposome with bilayer lipid, multilayered liposomes sometimes have better stability and performance in the sustained release (Jeon et al., 2015).

Cholesterol normally plays a compulsory component to stabilize the liposome (Nakhaei et al., 2021); however, it might induce pulmonary hypertension (Szebeni et al., 2000). Ginsenoside Rh2 conjugated to liposome (Rh2-lipo), not only replaces the cholesterol and PEG to stabilize the liposome and prolong its half-life in the blood circulation, but also acts as an active-targeting ligand, and chemotherapy adjuvant (Hong et al., 2020a).

Although lipids are essential for almost all liposome construction, lipid-free liposome has also been reported, depending on the amphiphilic nature of the drug with both hydrophilic and hydrophobic groups. For example, hydrophilic **resveratrol** was conjugated to hydrophobic norcantharidin (a derivative of **cantharidin**). The resveratrol-norcantharidin liposomes were prepared by ethanol solution injection and sonication method, and then spontaneously transformed to a tadpole-like structure after 24 h due to the small hydrophobic moiety and bulky NCTD moiety (Yan et al., 2016). This formulation not only improved the drug encapsulation, but also increased the toxicity in zebrafish embryos, compared with free monomer. This strategy helps to deliver natural products by combining hydrophilic and lipophilic molecules.

The payloads of liposomes included hydrophilic or hydrophobic natural product molecules, nanoparticles, and macromolecules (Rahman et al., 2012) as sub-container of natural products.

### Functional Liposomes

Traditional liposomes often have limitations in the low stability in physiological environments against self-aggregation and fast body clearance, low efficiency in targeted delivery, and controllable release. Hence, besides normal lipid as the major components regulating their size, stability and rigidity, additional functional lipids are essential for various purposes, such as PEG-lipid, ligand-lipids, antibody-lipid, unsaturated lipid, and thermo-sensitive lipid.

#### Long Circulation Liposome

As previously mentioned, PEGylated liposomes containing PEG-lipid are commonly used in clinical and other trials due to the increased water-solubility and the resistance to body clearance (Allen et al., 1995; Sercombe et al., 2015; Deodhar and Dash, 2018). The hydrophilic side of PEG-lipid on the surface of liposomes significantly increased the stability in water by hydration, compared with conventional liposomes which tend to aggregate in the aqueous phase. The sterically-stabilized liposomes by PEGylated modification also reduced the macrophages of the reticuloendothelial system (RES).

#### Activatable Liposomes

The activatable liposomes are capable of releasing cargoes in the lesion upon the external stimulus, such as temperature, sonic radiation, light, pH, ROS, and enzyme, which are normally responsible for site-selectively triggering the drug release.

For thermo-sensitive liposomes, the melting phase transition temperature (*T*_m_) of the lipids is the key to inducing the collapse of liposome and drug release. An ideal *T*_m_ is above the physiological temperature (37°C) of human body, which maintains the liposome stability to avoid unwanted leakage. However, it should not be too high to avoid the liposomes to be broken. For example, a K237-modified thermos-sensitive liposomes (K237-PTX-TSL) with DPPC of *T*_m_ (41.4°C) released 72.45% of **paclitaxel** in 20 min (Fu et al., 2019a).

For pH-sensitive liposomes, the liposomes were destabilized and released drugs (**doxorubicin** or **paclitaxel**) upon the pH-sensitive lipid DOPE of liposomes changed under low pH (Monteiro et al., 2018; Li et al., 2019). The free amine groups on DOPE molecules would obviously change their supramolecular organization when amine groups were acidified.

For light-/ROS-sensitive liposomes, upon light irradiation, the liposomal photosensitizer Ce6 induced the generation of ROS and oxidized the unsaturated lipid (egg yolk lecithin, PC-98T) to trigger the carrier collapse and release the co-encapsulated drug **triptolide** (Yu et al., 2021).

For sono-sensitive liposomes, it was believed that ultrasonic induced thermal effect and mechanic stimulation to result in lipid membrane pore and therefore release the drugs (**doxorubicin** and others) (Patrucco and Terreno, 2020).

#### Active-Targeted Liposomes

Over-expressing receptors are usually validated as a biomarker of many diseases, which have played very important roles in the drug delivery. The liposomes could target the lesion (e.g., tumor) specifically with the aid of antibody-antigen or ligand-receptor interactions.

Vascular endothelial growth factor (VEGF) monoclonal antibody (mAb) was conjugated to liposomes encapsulating **paclitaxel**, which selectively guided the delivery and accumulation in the VEGF-overexpressing tumor and release the chemotherapeutic agent within the tumor (Shi et al., 2015).

Ligand-conjugation on the surface of liposomes is another strategy for active targeting to enhance therapeutic effects by receptor-mediated endocytosis. Due to the diversities of biological activities, many natural products have shown strong affinities and specificity to the corresponding receptors, and therefore endow the liposomes with excellent recognition, such as **glycyrrhetinic acid** (**Glycyrrhizin**) (Mao et al., 2003; Mao et al., 2007; Li et al., 2012a; Tian et al., 2014; Cai et al., 2016), glycyrrhetinic acid derivative ligand 18-GA-Gly (Jin et al., 2017), **glycyrrhetinic acid** plus galactosylate (Chen et al., 2017), galactosylated-stearate (Li et al., 2018a), **galactose** (Guo et al., 2011), and **folate** (Chen et al., 2016; Guo et al., 2022). For example, **glycyrrhetinic acid** isolated from *Glycyrrhiza glabra* was used to modify liposome for liver targeting (Tsuji et al., 1991; Tian et al., 2014). **Glycyrrhetinic acid**-modified liposomes encapsulating **wogonin** (GA-WG-Lip) have better inhibition efficiency (IC_50_ 2.292 μM) to HepG2, compared with unmodified liposomes WG-Lip (IC_50_ 3.344 μM). GA-WG-Lip also improved cellular uptake and tumor recognition. The other ligands also showed good targeting and were modified on the liposome to deliver natural products, such as tLyp-1 (sequence CGNKRTR) (Jin et al., 2018), transferrin (Leto et al., 2016), and monosaccharide GalNAc with α configuration (Tn antigen) (Li et al., 2021a).

The combination of active-targeting elements is a popular approach to further enhance the delivery, e.g., antibody and tumor lineage-homing cell-penetrating peptide (Lin et al., 2018).

#### Biomimic Liposomes

Liposomes are vulnerable to body clearance, while exosomes have promising advantages including low immunogenicity, high bioavailability and targeted delivery, and so on, because of the similar structure of bilayer lipid, proper nanosize, and abundant proteins/receptors on the surface (Zhao et al., 2020; Chen et al., 2022). Exosome-liposome hybrid nanoparticles delivered **triptolide** to cisplatin-resistant ovarian cancer (Li et al., 2022). The CD47 and signal regulatory protein alpha (SIRPα) which overexpressed on exosomes derived from tumor cells, helped the nanoparticles to escape from the clearance of the mononuclear phagocyte system (MPS). **Triptolide** could be directly encapsulated into SKOV3-exosomes (SK-Exos) (Liu et al., 2019).

### Liposomal Medicinal Natural Products

We only picked up some typical examples from lots of liposomal natural products for the demo (Table 1).

**TABLE 1:** Liposomal natural products and their derivatives.

Drug	Source	Lipid composition	Size/nm	Bioactivity	Ref
Artemisinin	*Artemisia annua L*	Free	N.A.	*C*_max_: 0.25 ± 0.08 μM; AUG_0–24h_: 0.132; *t*_1/2β_: 0.38 h; CL: 63.11 ml/h	Isacchi et al. (2011a)
		P90G, CHOL	136.2 ± 42.27	*C*_max_: 1.11 ± 5.60 μM; AUG_0–24h_: 0.836; *t*_1/2β_: 0.67 h; CL: 9.97 ml/h	
		PEG2000, P90G, CHOL	132.6 ± 8.78	*C*_max_: 1.04 ± 0.01 μM; AUG_0–24 h_: 0.899; *t*_1/2β_: 2.02 h; CL: 9.20 ml/h	
		L-α-phosphatidylcholine, CHOL	79 ± 5	IC_50_: 6.0 ± 1.4 μg/ml for intracellular *Leishmania donovani* amastigotes; IC_50_: 5.1 ± 0.9 μg/ml for infected macrophages	Y. Want et al. (2017)
ADP109	Artemisinin	Free	N.A.	IC_50_: 0.07 ± 0.01 μM to BT474 cells; IC_50_: 10 ± 3 μM to MDA-MB231 cells	Zhang et al. (2013a)
		EPC	70 ± 20	IC_50_: 0.08 ± 0.01 μM to BT474 cells; IC_50_: 7 ± 2 μM to MDA-MB231 cells	
AMPm109	Artemisinin	Free	N.A.	IC_50_: 1.3 ± 0.8 μM to BT474 cells; IC_50_: >> 100 μM to MDA-MB231 cells	Zhang et al. (2013a)
		EPC	50 ± 20	IC_50_: 1.3 ± 0.4 μM to BT474 cells; IC_50_: >> 20 μM to MDA-MB231 cells	
Baicalin	*Scutellaria Scutellaria baicalensis Georgi*	HSPC, CHOL, F-PEG-CHEMS	68.4 ± 3.6	IC_50_: 58.3 ± 3.3 μg/ml to HeLa Cells	Chen et al. (2016)
		HSPC, CHOL, PEG-CHEMS	70.9 ± 1.4	IC_50_: 76.1 ± 4.6 μg/ml to HeLa Cells	
		HSPC, CHOL	87.6 ± 1.6	IC_50_: 78.8 ± 4.2 μg/ml to HeLa Cells	
		Free	N.A.	IC_50_: 64.6 ± 3.8 μg/ml to HeLa Cells	
BDMC	*Turmeric*	D-α-tocopherol polyethylene glycol 1000 succinate (TPGS or vitamin E TPGS), CHOL, mixlecithin	75.98 ± 5.46	Effective analgesic activity due to prolonged latency time, and the significantly decreased level of uric acid	Wang et al. (2021)
Betulinic acid	*White birch*	Egg phosphatidylcholine, egg phosphatidylglycerol	1000–1500	Efficiently inhibited human colon and lung tumors in nude mice	Mullauer et al. (2011)
Borneol	*Dryobalanops aromatica Gaertn f.*, *Blumea balsamifera DC*	Phospholipid, CHOL	167.1	BO upregulates drug effect and synergistically help the BA to promote the recovery of the brain by inhibiting neuronal cell damage and apoptosis	Zhang et al. (2020)
Breviscapine	*Erigeron breviscapus*	HSPC: CHOL, Pluronic P85	118.8 ± 4.9	Significantly increased absorption in Caco-2 cells and oral bioavailability in rats	Zhou et al. (2014)
		PC, PG, CHOL, triolein or tricaprylin	17,900	bre-MVL significantly prolonged the retention both *in vitro* and *in vivo* compared with those of bre-TL.	Zhong et al. (2005)
		PC, CHOL	540		
BP	*Angelica sinensis*	LPPC	200–280	Liposomal BP showed higher cytotoxicity to B16/F10 melanoma cells than free BP by arresting cell cycle at G0/G1 phase	Gao et al. (2018)
Camptothecin (CPT)	O*riental tree, amptotheca acuminata*	Single/two/three components of EPC, DOTAP, DPPG, DLPC, DMPG, DOPC, DOPE, DMPE-DTPA, DPPC, DMPC, CHOL	30–35	Liposomal CPT improved the circulated time in mouse, compared with free CPT.	Flaten et al. (2013)
Chlorophyll	*Chimonanthus salicifolius*	Soybean lecithin, CHOL	21.7 ± 6.0	Significantly improved the water solubility of lipophilic chlorophyll and its NIR fluorescence	Chu et al. (2012)
Curcumin	*Turmeric*	Free	N.A.	EC_50_: 1.9 and 1.5 μM to colon and lung cancer cells, respectively	Feng et al. (2017)
		EggPC, CHOL, DSPE-PEG	∼420	EC_50_: 0.96 and 0.90 μM to colon and lung cancer cells, respectively	
		EggPC, CHOL, DSPE-PEG (βCD-C)	∼420	EC_50_: 3.25 and 2.9 μM to colon and lung cancer cells, respectively	
Curcumol	*Rhizoma zedoariae*	Yolk lecithin, CHOL	<200 nm	Gal-s modified liposome enhanced the targeted delivery and treatment to liver cancer cells	Li et al. (2018a)
Daunorubicin	*Streptomyces peucetius*	DSPC, CHOL	45	Improved plasma half life and uptake by tumor	O'Byrne et al. (2002)
Docetaxel	*Glycyrrhiza glabra L. (licorice)*	Soybean phospholipids, CHOL	∼90 nm	GA-modified liposomes enhanced hepatocytes-target cellular uptake by receptor-mediated endocytosis	Li et al. (2012a)
Doxorubicin	*Streptomyces peucetius*	HSPC, CHOL, MPEG-DSPE	<100	AIDS-related Kaposi’s sarcoma; ovarian cancer resistant to paclitaxel and platinum	Waterhouse et al. (2001)
EPS	*Epimedium*	Soybean phospholipid, CHOL, tocopherol	200	The liposomal EPS significantly improved the immune response to Newcastle disease vaccine	Gao et al. (2012)
Ganoderic acid	*Lucid ganoderma*	P90G	150–180	Liposomal ganoderic acid has shown better antitumor effects by inhibiting various signaling pathways	Rahman et al. (2019)
Ginsenoside	*ginseng*	EYPC, CHOL	60.54 ± 1.78	The three ginsenoside-modifications enhanced the cellular uptake mediated by GLUT carriers	Hong et al. (2019)
			52.02 ± 1.42		
			99.02 ± 2.55		
Hirudin	*Hirudo spp*	DSPC, BC	191.49 ± 3.67	Inhibited the expression of VEGF and TGF-β1 in the rat kidneys	Wang et al. (2019)
Honokiol	*Magnolia species*	PC, cholesterol, PEG4000	130 ± 20	Liposomal honokiol significantly suppressed Lewis lung carcinoma overexpressing VEGF-D by inhibiting the tumor-associated lymphangiogenesis and metastasis	Wen et al. (2009)
Idarubicin	—	DSPC, DSPE-PEG2000	100 ± 30	Enhanced the circulation longevity of idarubicin to improve antitumor activity	Dos Santos et al. (2005)
Juglone	*Juglans mandshurica*	DPPC, CHOL	187 ± 12	The temperature-sensitive liposomes significantly inhibited HepG2 cell growth and proliferation upon exposed to hyperthermia	Zhao et al. (2016)
			220 ± 32		
LBP	*L. barbarum*	Soybean phospholipids, CHOL	121.5 ± 0.2	The LBPL-OVA vaccine formulation enhanced immune responses	Bo et al. (2017)
			121.13 ± 0.37		
Matrine	*Sophora flavescens*	HSPC, CHOL, DSPE-mPEG2000, DSPE-PEG-MAL, *c*RGD-SH	97.59 ± 1.93	RGD-M-LCL significantly improved the tumor-specificity and suppressed the proliferation of Bcap-37, HT-29 and A375 cells, compared with matrine alone	Liu et al. (2010)
Morphine	*Papaver somniferum*	HSPC, mPEG-DSPE, CHOL	120.45 ± 10.53	Prolong analgesic effect and reduce drug addiction	Gómez-Murcia et al. (2019)
OP	*Ophiopogonis japonicus*	Soybean phospholipid, CHOL	245.3	Liposomes significantly improved the immune-enhancing activity of OP on Kupffer cells	Fan et al. (2014)
		Soybean phospholipid, CHOL	245.3	Liposomes significantly improved the immune-enhancing activity of OP against Newcastle disease virus on chicken	Fan et al. (2015)
		Soybean phospholipid, CHOL	245.3	Liposomal OP significantly activated mouse peritoneal macrophages	Sun et al. (2016)
		Soybean phospholipid, CHOL	245.3	Liposomal OP significantly enhanced the antioxidative and immunoregulatory activities of OP in ICR mice	Fan et al. (2016)
Paclitaxel	*Taxus chinensis*	Free	N.A.	IC_50_: 42.38 ± 2.4 and 14.71 ± 1.37 nmol/L to SKOV-3 cells and HUVECs, respectively	Fu et al. (2019a)
		DPPC, DSPG, MPPC, DSPE-PEG	80.2 ± 3.9	IC_50_: 31.19 ± 2.02 and 11.51 ± 1.13 nmol/L to SKOV-3 cells and HUVECs, respectively	
		DPPC, DSPG, MPPC, DSPE-PEG, DSPE-PEG-K237	88.3 ± 4.7	IC_50_: 13.61 ± 1.81 and 5.54 ± 0.95 nmol/L to SKOV-3 cells and HUVECs, respectively	
		DSPE-PEG2000-Tn, DPPC	74 ± 0.36	IC_50_: 1.93 nM to HepG-2 cells	Li et al. (2021a)
Parthenolide	*Tanacetum parthenium*	SPC	118.6 ± 0.2	PTL-Lips enhanced the antitumor efficacy	Gao et al. (2020a)
PPa	Terrestrial and marine plants, insect fluid	DPPC, CHOL, DSPE-mPEG_5k_	100	Significantly improved the water solubility, prolong blood circulation and the bio-distribution in mice	Zhou et al. (2019)
PpIX	Living cells	PC	124 ± 0.85	IC_50_: 0.53 ± 0.19 µM to Hela cells	Przybylo et al. (2016)
RGP	*R. glutinosa*	Soybean phospholipid, CHOL, Tween-80	170.83 ± 2.08	The positive modulation effects on dendritic cells	Huang et al. (2016)
			193.57 ± 1.89		
Resveratrol	*Polygonum cuspidatum decoction pieces*	Soybean lecithin, CHOL	146–585	Liposomal resveratrol showed neuroprotective effects on mitochondria in substantia nigra cells of Parkinsonized rats	Chen et al. (2021)
Sal B	*Salvia miltiorrhiza Bge*	PEG2000(18:0/18:0), P90G, CHOL	140.0 ± 6.5	The PEGylated liposome improved the antihyperalgesic effect by prolonged time	Isacchi et al. (2011b)
Topotecan	Synthetic analog of camptothecin	Sphingomyelin, CHOL	100 ± 20	The formulation enhanced the half-life in plasma	Chernov et al. (2017)
Triptolide	*Tripterygium wilfordii*	TRX-20, HSPC, PEG5000-PE	117.9 ± 1.4	Significantly improved the anti-inflammatory effects in membranous nephropathic model	Yuan et al. (2017)
Vincristine	*Catha-ranthus roseus*	DSPC, CHOL	100	Prolonged retention time dramatically improved the therapeutic effects on mice bearing P388 murine leukemia	Boman et al. (1994)
Wogonin	*Scutellaria baicalensis Georgi*	Free	NA	IC_50_: 16.248 mg ml^−1^ to HepG2 cells	Tian et al. (2014)
		Soybean phospholipids, CHOL	87.4 ± 4.8	IC_50_: 3.344 mg ml^−1^ to HepG2 cells	
		Soybean phospholipids, CHOL, 18-GA-Suc	90.5 ± 2.2	IC_50_: 2.292 mg ml^−1^ to HepG2 cells	

**FIGURE 2:**
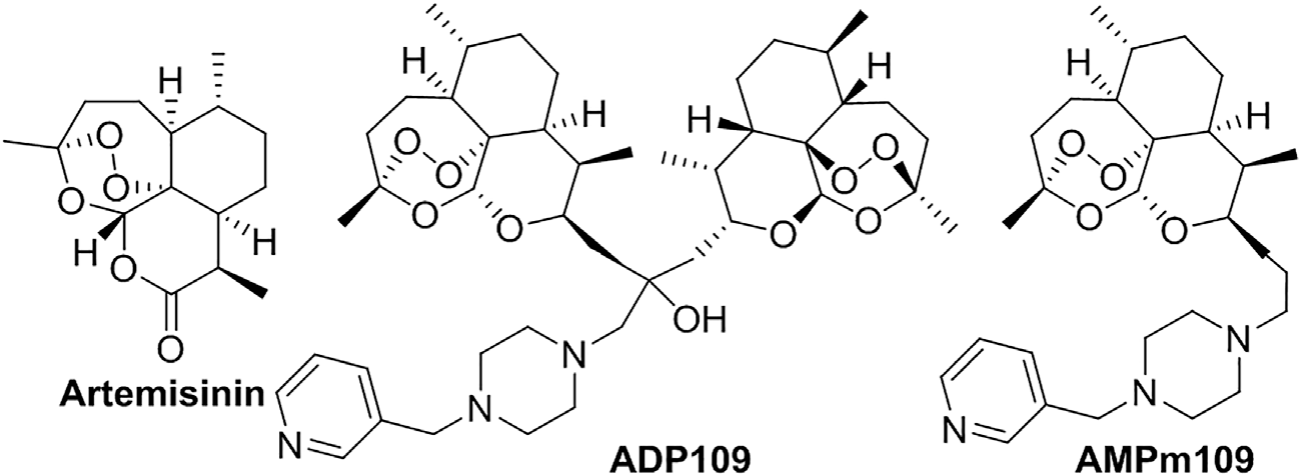
Liposomal **artemisinin** and its analogs.

**Artemisinin**, isolated from *Artemisia annua L.*, was discovered in 1972 to treat malaria by Youyou Tu who was awarded the Nobel Prize in physiology or medicine (Figure 2) (Isacchi et al., 2011a; Memvanga and Nkanga, 2021). It also showed activities in chemotherapeutic drug for tumors and lupus erythematosus (Mu and Wang, 2018). The sensitive endoperoxide bridge is believed to be essential for its antimalarial activity. However, this drug always suffers problems in low stability, poor water-solubility, short-duration effect, short half-life and high first-pass metabolism, and so on. Compared with free drugs, both liposomal **artemisinin** (conventional (A-L) and PEGylated (A-PEGL) liposomes), obviously increased the pharmacokinetic parameters, such as the peak concentration (C_max_), the area under the plasma concentration versus the time curve (AUC_0–24h_), and half-life (t_1/2β_) (Isacchi et al., 2011a). Two pH-responsive artemisinin derivatives, ADP109 and AMPm109 were conjugated to piperazine moiety (Zhang et al., 2013a). Due to the modification, their water-solubility was enhanced at acidic pH, which is responsible for the site-specific drug release from liposomes. Liposomal **artemisinin** also improved its anti-leishmanial potential, compared to its free form (Y. Want et al., 2017).

**FIGURE 3:**
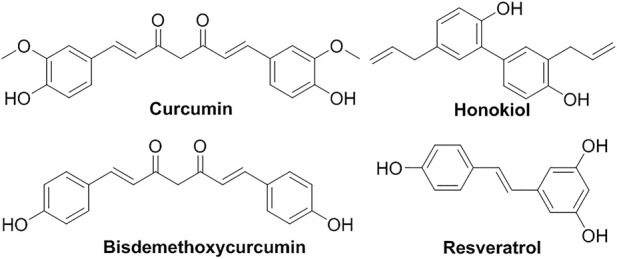
Liposomal polyphenolic compounds.

Polyphenolic compounds are very common secondary metabolites in plant against ultraviolet radiation and pathogens (Figure 3) (Pandey and Rizvi, 2009). For example, **resveratrol** liposomes showed protective effects on mitochondria in substantia nigra cells of parkinsonized rats (Chen et al., 2021). Liposomal **honokiol** has shown various biological activities (Niu and Gao, 2009; Guillermo-Lagae et al., 2017; Ong et al., 2020; Li et al., 2021b; Li et al., 2021c). Liposomal **honokiol** significantly suppressed Lewis lung carcinoma overexpressing VEGF-D by inhibiting the tumor-associated lymphangiogenesis and metastasis (Wen et al., 2009). In 2017, Zhao et al. comprehensively reviewed a variety of liposomal formations of **curcumin** and its bio-application, which demonstrated its limitation in the poor water-solubility, low bioavailability and rapid body clearance and how liposome formulation significantly improved the inhibition of cancer cells (Rahman et al., 2012; Feng et al., 2017). The vitamin E TPGS-conjugated liposome formulation of poor soluble **bisdemethoxycurcumin** (**DBMC**) has significantly enhanced its stability, solubility, and bioavailability (Wang et al., 2021).

**FIGURE 4:**
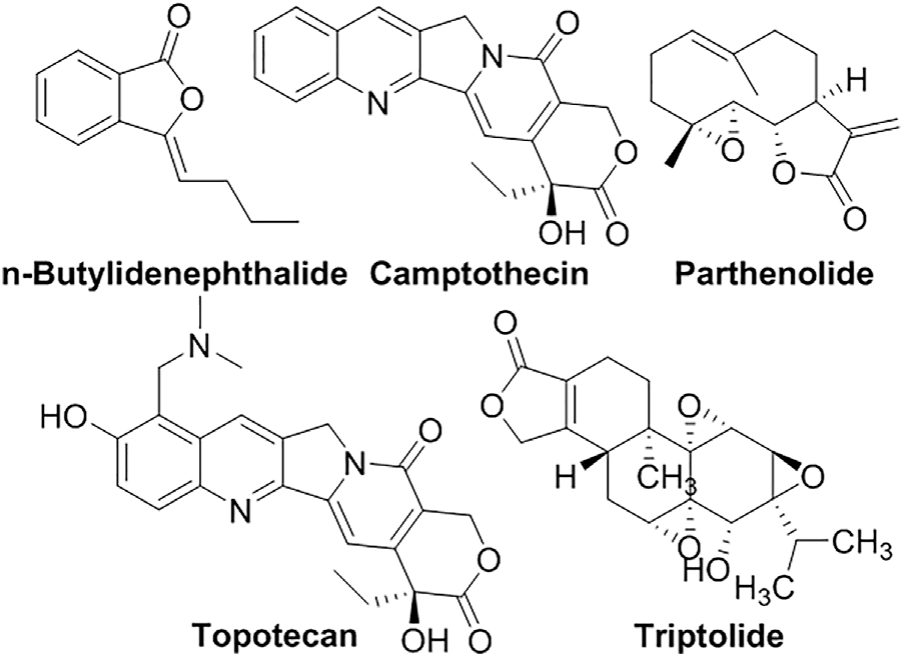
Liposomal lactones.

Lactones are cyclic carboxylic esters, typically with 5- or 6-membrane-ring, making them very active to be hydrolyzed or attacked by nucleophiles (Figure 4). This poor stability makes it difficult to tolerate physiological environments. **Camptothecin** (**CPT**) isolated from *Camptotheca acuminata* (Camptotheca, Happy tree) (Wall et al., 1966), which used in TCM or Chonemorpha fragrans, commonly used in Ayurveda (an Indian root medicine) (Govindachari and Viswanathan, 1972), has been used to treat tumor as a topoisomerase inhibitor. Its lactone form is active while the hydrolyzed/ring-opening form is inactive. To improve its poor aqueous solubility, and avoid hydrolysis or protein interactions, **CPT** was encapsulated into liposomes, as well as its other analogs, such as **topotecan**, **irinotecan**, **lurtotecan,** and **belotecan** (Emerson, 2000; Flaten et al., 2013). For example, liposomal **topotecan** was used to treat advanced malignancies and neuroblastoma (Zamboni et al., 2009; Chernov et al., 2017). n-**Butylidenephthalide** (**BP**) isolated from *Angelica sinensis* was encapsulated into a polycationic liposome for the treatment of B16/F10 melanoma cells (Gao et al., 2018). 3,5-Dipentadecyloxybenzamidine hydrochloride (TRX-20)-modified liposomes encapsulating **triptolide** significantly improved the anti-inflammatory effects in the membranous nephropathic model (Yuan et al., 2017). Due to the scaffold of *α*-methylene-*γ*-lactone, **parthenolide** has high electrophilicity to alkylate proteins and thus processes various bioactivities (Jin et al., 2018; An et al., 2022). pH-sensitive liposomes PTL-Lips encapsulating **parthenolide** have enhanced its antitumor efficacy (Gao et al., 2020a).

**FIGURE 5:**
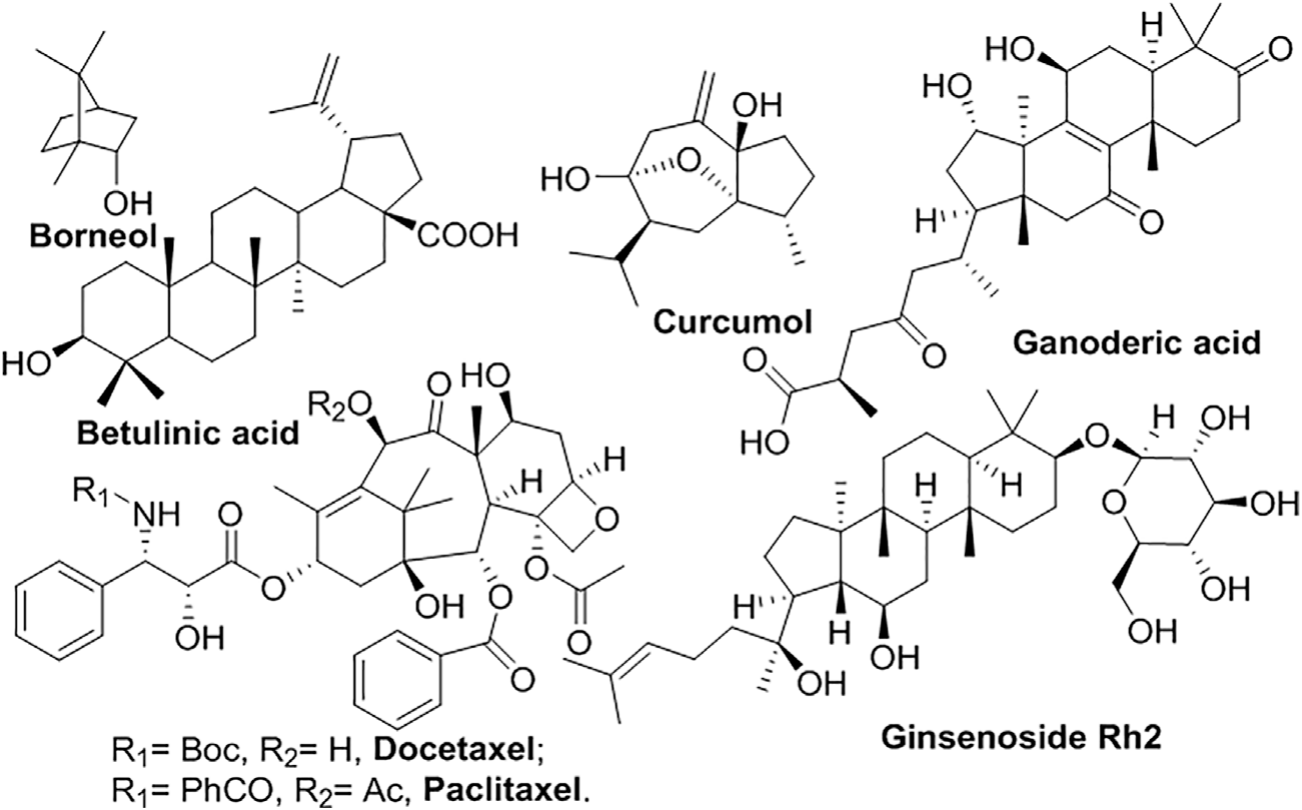
Liposomal terpenoids.

Terpenoids are derived from a number of isoprenes and possess functional groups (at least -OH), classified as monoterpene, diterpenoids, sesquiterpenoid, triterpenoids, and so on (Figure 5) (Ludwiczuk et al., 2017). Generally, they are hydrophobic, which means low water solubility. **Borneol**, a bicyclic monoterpene, isolated from *Dryobalanops aromatica Gaertn f.* and *Blumea balsamifera DC*, has been used as a messenger drug encapsulated in liposomes to help other drugs penetrate physiological barriers, such as blood-brain barrier (Zhang et al., 2020; Li et al., 2021d). Galactosylated-stearate (Gal-s) modified liposome enhanced the targeted delivery of **curcumol** and the treatment of liver cancer cells (Li et al., 2018a). Liposomal **ganoderic acid** has shown better antitumor effects by inhibiting various signaling pathways (Rahman et al., 2019). Liposomal formulation of **betulinic acid** enhanced the antitumor effects and reduced systemic toxicity (Mullauer et al., 2011; Zhang et al., 2016). Taxanes with a tetracyclic diterpene, including **paclitaxel** (Taxol^®^) and its synthetic analog **docetaxel** (Taxotere^®^) have shown antitumor activity by inhibition of microtubules and cell division. The temperature-sensitive liposomes (PTX-TSL) modified with K237 peptide enhanced the cellular uptake and cytotoxicity of **paclitaxel** against cancer cells and endothelial cells (Fu et al., 2019a; Li et al., 2021a). *N*-acetylgalactosamine modified liposome (Tn-Lipo-PTX) improved the targeted cytotoxicity to HepG2 cells. **Docetaxel** (Taxotere) was specifically delivered to hepatocytes by **glycyrrhetinic acid**-modified liposomes *via* receptor-mediated endocytosis (Li et al., 2012a). **Glycoside** normally contained a sugar bound to another functional group *via* a glycosidic bond (Li et al., 2018b). For example, **ginsenoside** (triterpenoid glycoside), isolated from *genus Panax* (*ginseng*), a class of triterpene **glycosides** (Hong et al., 2019). The liposomes Rh2-lipo derived from **ginsenoside Rh2** have shown multifunction in drug delivery, compared with conventional liposomes (Hong et al., 2020b).

**FIGURE 6:**
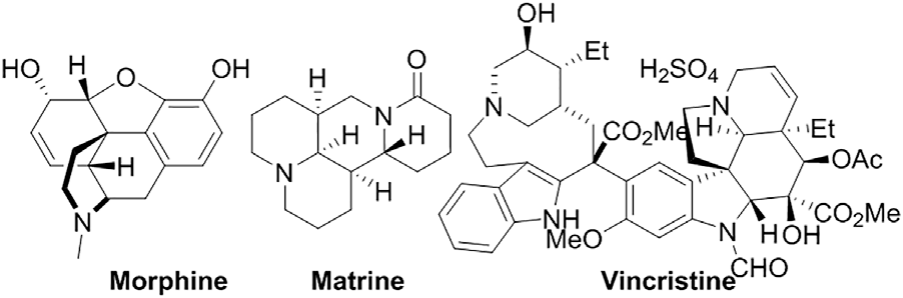
Liposomal alkaloids.

Natural alkaloids normally are basic, contain at least a nitrogen atom, and exhibit diversified biological activity as the secondary metabolites (Figure 6). Their free base form normally has poor water solubility, while their salts are soluble in water (Kukula-Koch et al., 2017). **Morphine**, isolated from *Papaver somniferum*, has been universally used to ease the pain. However, it is highly addictive because the high concentration accesses the central nervous system, which normally occurs when the free form is administrated. Liposome formulations prolonged the analgesic effect and reduce addiction (Gómez-Murcia et al., 2019). The formulation also significantly enhanced antitransit effects of morphine (Pol et al., 1996). **Matrine**, isolated from *Sophora flavescens*, used to treat hepatic diseases, has shown anti-tumor activity with fewer side effects, compared with other chemotherapeutic drugs. However, its low efficiency of tumor-targeting delivery and moderate anti-cancer activity hindered its further application. Liposomal **matrine** inhibited the growth of brain glioma (Han et al., 2014). The RGD-modified liposomes loading **matrine** (RGD-M-LCL) significantly improved the tumor-specificity and suppressed the proliferation of Bcap-37, HT-29 and A375 cells, compared with the free **matrine** (Liu et al., 2010). **Vincristine**, an antitumor natural alkaloid isolated from *Catharanthus roseus*, was encapsulated in liposomes (Marqibo^®^) (Pathak et al., 2014). A ganglioside G_M1_ incorporated liposome dramatically prolonged the retention time of **vincristine** and therefore improved the therapeutic effects on the mice bearing P388 tumor (Boman et al., 1994).

**FIGURE 7:**
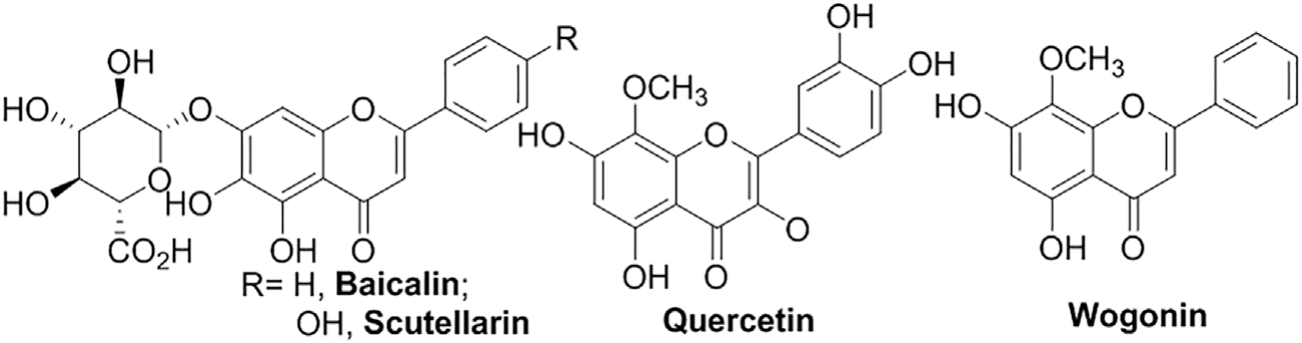
Liposomal flavonoids.

Flavonoids share the backbone of 2-phenyl-1,4-benzopyrone, which normally exhibit fluorescence under UV irradiation and poor solubility in water (Figure 7) (Panche et al., 2016). Liposomal **quercetin** significantly improved the accumulations in tumor and circulation time, and inhibited tumor growth (Yuan et al., 2006). The antioxidant **quercetin** encapsulated in liposomes also showed the potential to reduce the oxidative damage to hepatic tissue caused by CCl_4_ (Mandal and Das, 2005). **Breviscapine** (**Scutellarin** as the main component), isolated from TCM *Erigeron breviscapus*, was extensively used to treat ischemic cerebrovascular and cardiovascular diseases in China, however, it suffered short circulation time (Zhong et al., 2005; Zhou et al., 2014). The drug durations both *in vitro* and *in vivo* were significantly prolonged by multivesicular liposome formulation (Zhong et al., 2005). Compared with traditional liposomes releasing 80% of payload within only 4 h *in vitro*, multivesicular liposomes have shown a better sustained-delivery system for **breviscapine** which extended the period to 5–6 days. **Baicalin** (Chen et al., 2016; Xiang et al., 2020) has poor solubility (91 μg/ml) in water at room temperature and oral bioavailability (2.2%). The folate-conjugated liposome-encapsulated **baicalin** has improved its cytotoxicity and cell uptake. **Wogonin** (**WG**), isolated from *Scutellaria baicalensis Georgi* as a TCM treating inflammation, has been explored with wide-spectrum therapeutic effects, such as anti-tumor, anti-oxidant, anti-hepatitis B virus, anticonvulsant and neuroprotective effects (Tian et al., 2014). The **wogonin** liposome (WG-Lip) significantly improved the bio-distributions, compared with **wogonin** solution. The **glycyrrhetinic acid**-modified liposomes encapsulated **wogonin** (GA-WG-Lip) further improved targeted delivery. The cytotoxicity test against HepG2 cells and *in vivo* anti-tumor efficacy also reflected the tendency.

**FIGURE 8:**
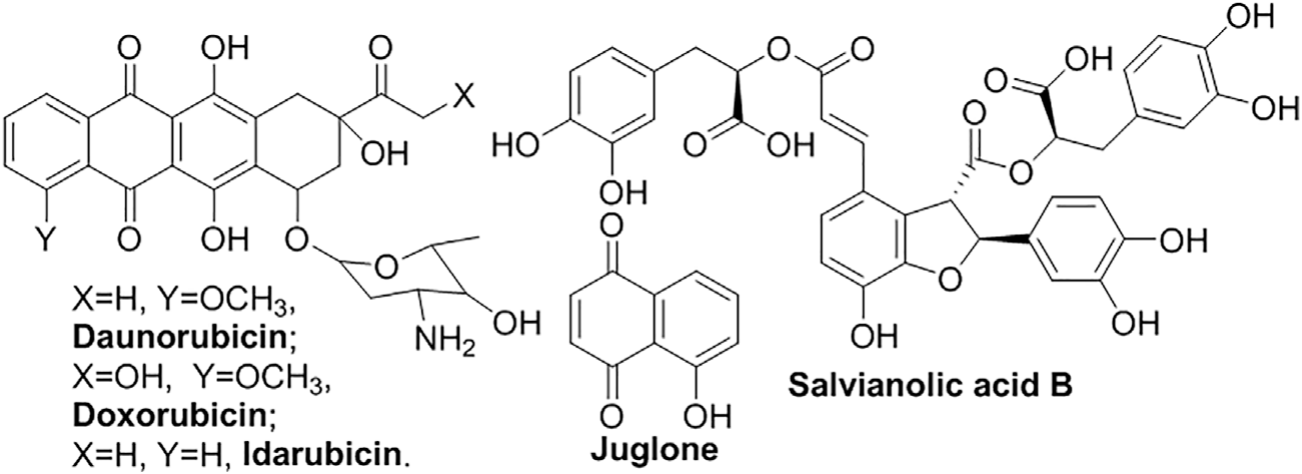
Other liposomal natural products.

Anthracyclines have a tetracyclic core with an anthraquinone backbone connected to a sugar moiety by a glycosidic linkage (Figure 8). They generally exhibit various anti-cancer effects by inhibiting topoisomerase II and the synthesis of DNA and RNA. For example, **daunorubicin**, and **doxorubicin**, isolated from bacteria of the *Streptomyces* type, were encapsulated in liposomes (DaunoXome^™^ and Doxil^®^) for cancer chemotherapy (Schmidt et al., 1998; Piccaluga et al., 2002; Abraham et al., 2005; Waterhouse et al., 2001; O'Byrne et al., 2002). Liposomal formulation enhanced the circulation longevity of **idarubicin** to improve antitumor activity (Dos Santos et al., 2005). Temperature-sensitive liposomes t-L encapsulating **juglone** guided the targeted therapy of HepG2 cancer cells when they were exposed to hyperthermia and released **juglone** (Zhao et al., 2016). **Salvianolic acid B** (**Sal B**) from *Salvia miltiorrhiza Bge* exhibits a strong antioxidant and free radical-scavenging activity to relieve oxidative stress (Isacchi et al., 2011b; Lin et al., 2014). The water-soluble compound, sensitive to UV irradiation and aqueous solutions, also suffers the limitations in poor instability and oral bio-availability. Compared with free and conventional liposomal **Sal B**, the PEGylated liposomes improved the anti-hyperalgesic effect for a prolonged time.

Polysaccharides are polymeric carbohydrates composed of repeating monosaccharide units linked by glycosidic bond, from linear to highly branched polymeric. Liposomal **ophiopogon polysaccharide** (**OP**) from *Radix Ophiopogonis Japonici* (a TCM), significantly improved the immune-enhancing activity of **OP** on Kupffer cells (Fan et al., 2014) and non-specific/specific immune response in chickens (Fan et al., 2015). The liposomes also activated mouse peritoneal macrophages (Sun et al., 2016). Liposomal **OP** improved anti-oxidative and immunological activities, compared with free form (Fan et al., 2016). Liposome formulation also improved **lycium barbarum polysaccharides** (**LBP**) (Bo et al., 2017) and **rehmannia glutinosa polysaccharide** (**RGP**) (Huang et al., 2016) as antigens for vaccine development. The liposomal **heparin** spraygel demonstrated comparable efficacy in the treatment of superficial venous thrombosis (Pleban et al., 2008). The liposomal **epimedium polysaccharide** (**EPS**) assisted in significantly improving the immune response to the Newcastle disease vaccine (Gao et al., 2012).

Bioactive natural peptides play vital roles in many cellular bioactivities and therefore are used to treat many diseases (Lau and Dunn, 2018). However, it also has limitations in low oral bioavailability and short half-life in plasma. Liposomal formulation was developed for oral administration of **insulin** (Değim et al., 2006). **Hirudin**, isolated from *Hirudo spp.*, is a naturally occurring peptide and is therefore used as an anticoagulant to treat many diseases, e.g., diabetic nephropathy (DN). However, the lack of lesion targeting may result in severe side effects, such as hemorrhaging. The liposome significantly enhanced the renal targeting delivery and accumulations, compared with free **hirudin**, which demonstrated the relief of the renal injury in the diabetic nephropathy rat model (Wang et al., 2019).

**FIGURE 9:**
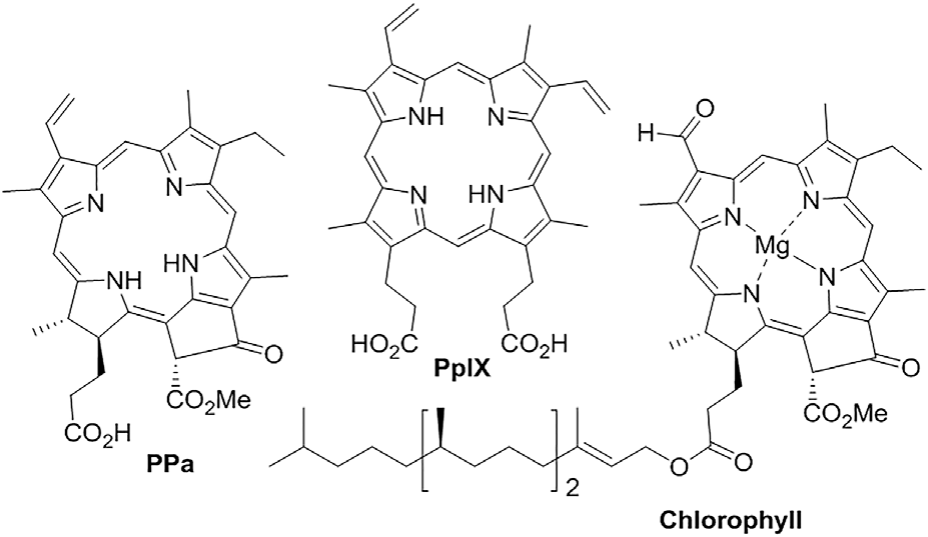
Liposomal porphyrinoids.

Porphyrinoids represent a group of macrocyclic tetrapyrrolic compounds with good photosensitivity, near-infrared (NIR) absorption and high fluorescent quantum yield, which grants the applications in photodynamic therapy (PDT), fluorescent imaging, and photothermal therapy (PTT) (Figure 9) (Liu et al., 2014). To solve their problems in poor water solubility and low stability in physiological environments, liposomal formulation significantly improved the delivery and uptake (Cheng et al., 2021). The lipid medium also disperses them to reduce their aggregation and self-quenching for better photo-sensitivity. **Pyropheophorbide acid** (**PPa**), a degraded product of chlorophyll, has been encapsulated into liposome for tumor PDT (Zhou et al., 2019). Under 690 nm-laser irradiation, liposomal **PPa** significantly suppressed tumor growth. The formulation of **protoporphyrin IX** (**PpIX**) improved its internalization and bio-distribution in HeLa cells (Przybylo et al., 2016). The liposomes have significantly improved the water solubility of lipophilic **chlorophyll** and its NIR fluorescence in sentinel lymph node mapping (Chu et al., 2012).

Betainylated cholesterol (BC), Egg phosphatidylcholine (PC), Phospholipon 90G (P90G), Polyethylene glycol (PEG2000), L-a-Phosphatidylcholine extracted from eggs (EPC), phosphatidylglycerol (PG), Polyethylene glycol complex (LPPC), 1,2-Dipalmitoyl-sn-glycero-3-phosphocholine (MPPC), 1,2-Distearoyl-sn-glycero-3-phospho-(10-rac-glycerol) (DSPG), 1-Myristoyl-2-palmitoyl-sn-glycero-3-phosphatidylcholine (MSPC), 1,2-distearoyl -sn-glycero-3-phosphoethanolamine-N-[PEG(2000)] (DSPE-mPEG2000), Malei-(ethylene glycol)]-1,2-dioleoyl-sn-glycero-3-phosphoethanolamine (DSPE-PEG-MAL), Dipalmitoylphosphatidylcholine (DPPC), Cholesterol (CHOL), 3-Succinyl-30-stearyl glycyrrhetinic acid (18-GA-Suc), Cholesteryl hemisuccinate (CHEMS), Hydrogenated soybean phosphatidylcholine (HSPC), Egg yolk lecithin (EYPC), Distearoyl phosphatidylcholine (DSPC), Monomethoxy polyethyleneglycol 2000-distearoyl phosphatidylethanolamine (mPEG-DSPE), Hydrogenated soy phosphatidylcholine (HSPC), Methoxy polyethylene glycol (MPEG), Distearoyl phosphatidylethanolamine (DSPE)

### Liposomal Natural Product Extract

Owing to the multi-components of natural products, TCM is believed to treat disease by multi-targets interactions, which is especially suitable for drug-resistant therapy. However, it is difficult to clearly and comprehensively demonstrate the key concerns of drug discovery, such as pharmacokinetics and side effects, although many signs of progress have been made to analyze their components, biological targets, metabolites, and signal transduction by applications of modern analytic methods for enzyme, gene and the interactions between them (Su et al., 2007). Although a single drug molecule with clear structure and pharmacokinetics is the mainstream of modern drug discovery, TCM or other herbal medicines containing a complicated mixture of natural products with unclear components and mechanism, have obtained many surprising achievements in various therapies for a long time, which are really hard to refuse.

Thus, to minimize the possible risks and maximize the therapeutic effects, liposomal formulation of the extracts or ingredients has been applied (Bilia et al., 2018). The extract of schisandra chinensis fructus (SCF, a TCM) from *Schisandra chinensis (Turcz.) Baill* was encapsulated in β-cyclodextrin (β-CD) and then liposome. By analyzing three major bioactive lignans (Schisandra lignans: **Schisandrin** (SD), **schisantherin** (ST), and **c-schizandrin** (SZ)), this formulation enhanced liver uptake (Ding et al., 2019). The liposomal formulation of *Aphanamixis polystachya* leaf extract showed better performance in the behavior of mice dementia model than the extract (Shariare et al., 2020). The liposomes have high encapsulation efficiency of poor water-soluble pollen extract and therefore improved the bioaccessibility (Hızır-Kadı et al., 2020). Using unpurified soybean phospholipids, the nano-liposomes encapsulating *Orthosiphon stamineus* ethanolic extract significantly improved the intestinal absorption and anti-oxidation effects (Aisha et al., 2014).

## Multifunctional Liposomes for Disease Treatment

Due to the flexibility in the surface modifications and big capacity for hydrophobic and hydrophilic cargoes, liposome has proved to be an excellent multifunctional platform for combined therapies and image-guided therapy. It may further improve the systematic therapeutic effects, compared with monotherapy by the medicinal natural products alone.

### Synergistic Combined Therapies

Single molecular treatment usually has a maximally tolerated dose, which probably limits the therapeutic effects. Furthermore, to survive against the treatment, cancer or pathogen has high probability to develop resistance as an “acquired” ability by the fast drug-clearance response and self-evolution, which usually occurs in single therapy, such as chemotherapy (Vasan et al., 2019), radiotherapy (Willers et al., 2013), immunotherapy (Bai et al., 2020), PDT (Casas et al., 2011), and so on. It is worthy to mention that resistant cancer or pathogen will become more aggressive and refractory. To solve the problems, combined therapy has shown promising therapeutic effects. The different drugs in the co-delivery liposomal system could simultaneously and synergistically block the distinct pathways involved in the survival of the targets, which makes the targeting cancer or pathogen more sensitive to the treatments. Therefore, the dose and side-toxicity of individual drugs might be reduced, as well as the possibility of drug resistance due to less exposure to the drugs.

#### Combined Chemotherapies

The chemotherapeutic drug suppresses cell proliferation and induces cell death by inhibiting cell survival activities. Combined chemotherapies obviously enhance the therapeutic effects and defeat drug resistance.

To treat drug-resistant malaria infection, **artemisinin** was co-encapsulated with **curcumin** into a liposomal system (Isacchi et al., 2012). In another case, stearylamine liposomal **monensin** was combined with free **artemisinin** to afford enhanced antimalarial effects which were significantly affected by stearylamine and length of PEG-lipids (Rajendran et al., 2016).

When **ginsenosides** (Rh2, Rg3, and Rg5) was combined with **paclitaxel** (PTX), **ginsenoside** functioned as not only a chemotherapy adjuvant but also a functional membrane material to stabilize liposome and assist active-targeting (Hong et al., 2019). This multifunctional additive helped liposomal system to offer a novel platform for drug delivery.

**Paclitaxel** was encapsulated in thermosensitive liposomes, and modified with a therapeutic peptide K237 (sequence: HTMYYHHYQHHL) which inhibited the bonding between VEGF and KDR, and the proliferation of human endothelial cells (Fu et al., 2019a). The combined therapy of **paclitaxel** and k237 enhanced cell uptake and cytotoxicity against cancer cells and endothelial cells.

tLyp-1-conjugated liposomes encapsulating **parthenolide** and **ginsenoside CK** enhanced the antitumor activity and reduced the side effects, compared with individual drugs (Jin et al., 2018).

Cisplatin is a first-line chemotherapeutic drug for cancer therapy. However, it is highly debatable because of the severe side effects/toxicity and drug resistance. In the combined therapy of EMT-6 and B16F10 tumor models, although less dose of free cisplatin, liposomal **curcumin** has improved the antitumor effects and reduced toxicity (Hamano et al., 2019). The combination of liposomal **honokiol** with cisplatin improved antitumor activity by enhanced induction of apoptosis and inhibition of angiogenesis in A549 lung cancer xenograft model (Jiang et al., 2008). The combination has also been applied to cisplatin-sensitive (A2780s) and -resistant (A2780cp) human ovarian cancer models, which showed significant inhibition (84–88%) and prolonged the survival life (Luo et al., 2008).

A synergistic therapy of lung cancer treatment was achieved by a cocktail of **betulinic acid**, **parthenolide**, **honokiol,** and **ginsenoside Rh2** in a liposomal delivery system (Jin et al., 2020). Additionally, the combined therapies were relatively safer, compared with the treatment by cisplatin which exhibited obvious kidney damage.

Although the combination of **daunorubicin** and **cytarabine** was the standard for acute myeloid leukemia (AML) in the past decades, a liposomal formulation (CPX-351, Vyxeos^™^) of the combination was newly approved by the US Food and Drug Administration (FDA) for therapy-related acute myeloid leukemia (t-AML) or AML with myelodysplasia-related changes (AML-MRC), which improved the effects to the subgroups with the reason unclear (Chen et al., 2018).

Liposome co-delivered not only the cytotoxic natural products with other drugs for anti-proliferation (e.g., anti-tumor, anti-malaria, and so on), but also natural products for promoting proliferation. For example, due to the roles in promoting endothelial cell repair and capillary blood circulation, liposomal **heparin** was further combined with ibuprofen in “nano-spray gel” which exhibited significant effects in wound healing for frostbite (Vaghasiya et al., 2019).

#### Chemotherapy with miRNA Therapy

Liposomes improved the targeted delivery and protected the unstable miRNA from the degradation induced by physiological environments and RNA enzymes (Rupaimoole and Slack, 2017; Fu et al., 2019b).

**FIGURE 10:**
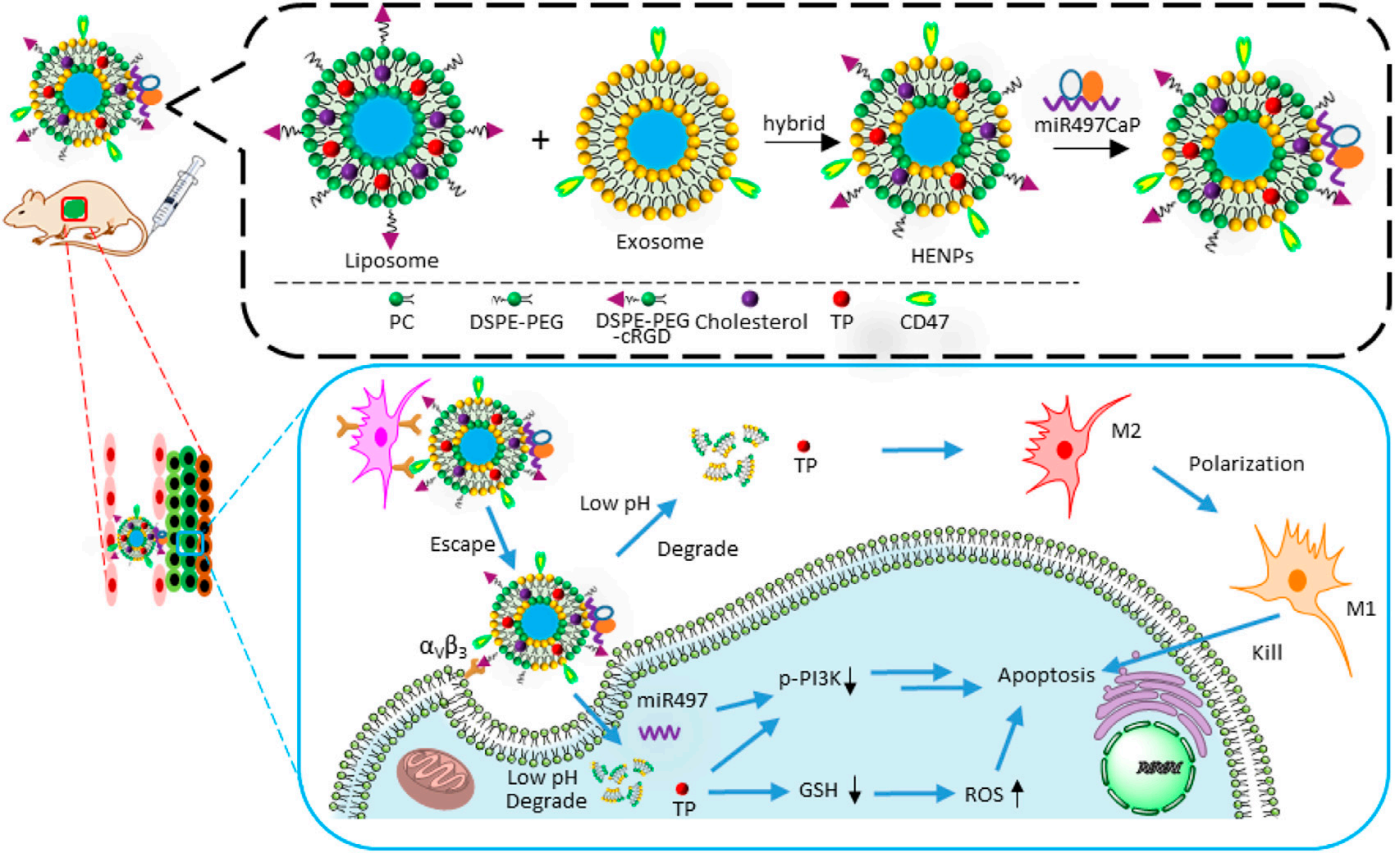
The preparation of miR497/TP-HENPs and the mechanism of combined therapies: chemotherapy with miRNA therapy. **Triptolide** (TP).

Bioinspired nanoparticles hybridizing CD47-expressing exosomes and cRGD-modified liposomes, encapsulated **triptolide** and miR497 to treat cisplatin-resistant ovarian cancer (Figure 10) (Li et al., 2022). After the NPs were delivered and accumulated in tumor cells by cRGD guidance and EPR effects, the acid-sensitive carriers encapsulating calcium phosphate (CaP), released the cargoes in the low pH tumor microenvironments. Meanwhile, CD47 helped the carrier to escape from the immune attack. The released **triptolide** and miR497 initiated the cancer cell apoptosis by multiple pathways. Firstly, **triptolide** efficiently promoted the polarization of M2 macrophages to M1 form. Secondly, **triptolide** upregulated cellular GSH and down-regulated ROS. Thirdly, it was believed that **triptolide** and miR497 synergistically inhibited PI3K/AKT/mTOR signaling pathway. In general, the combined therapies improved the antitumor effects and defeated the drug resistance.

#### Chemotherapy with PDT

In presence of light, oxygen and photosensitizer, PDT damages the targets with reactive oxygen species (ROS), exhibiting distinct advantages in almost non-invasion, low dark toxicity and low drug-resistance. Liposome has shown the excellent ability to combine PDT with chemotherapy and other therapies (Cheng et al., 2021).

**FIGURE 11:**
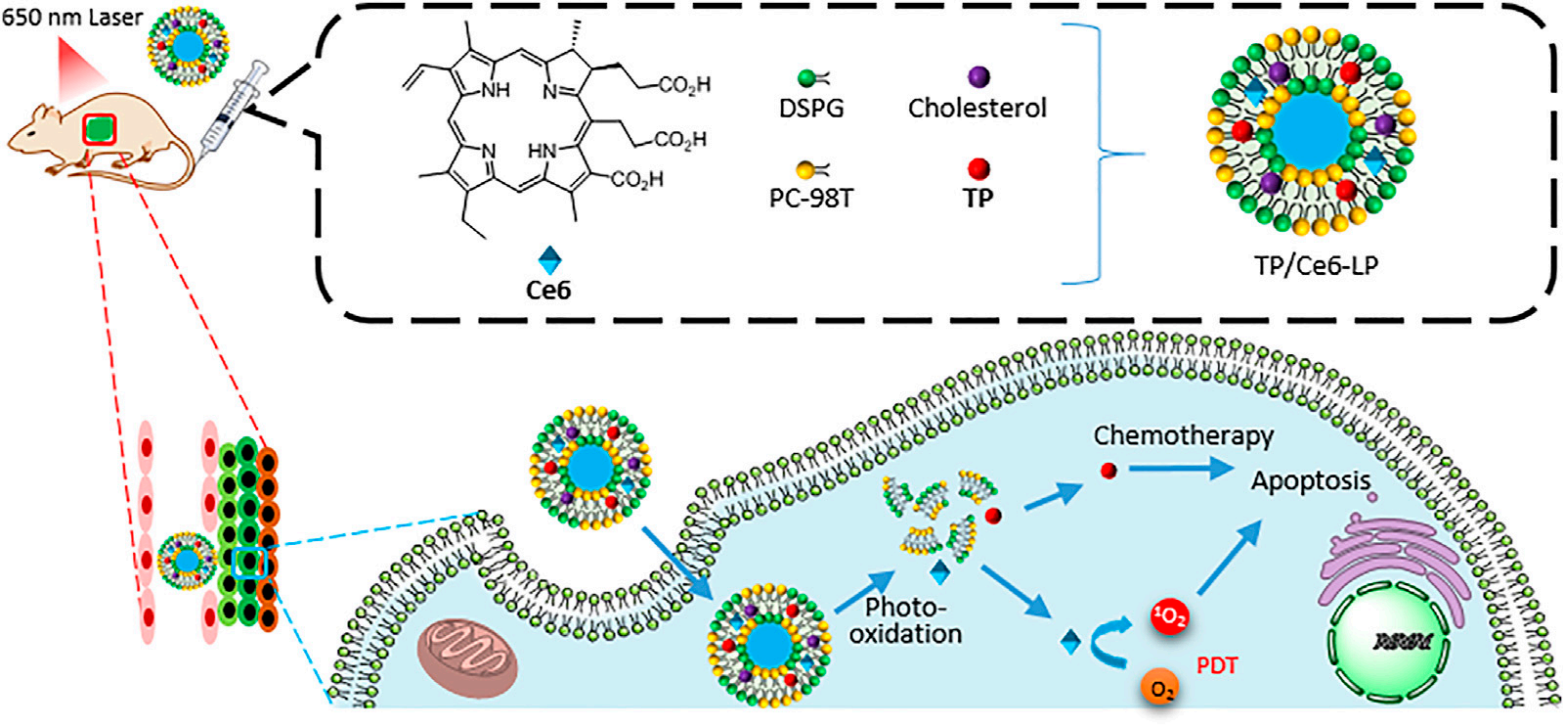
The preparation of TP/Ce6-LP and the mechanism of combined therapies: chemotherapy with PDT.

A photosensitive liposomal system TP/Ce6-LP, encapsulating natural product **triptolide** as a chemotherapeutic agent and Ce6 as a photosensitizer, was applied for hepatocellular carcinoma therapy (Figure 11) (Yu et al., 2021). Firstly, upon NIR irradiation (650 nm laser), Ce6 converted oxygen to ROS (^1^O_2_) to photo-oxidize the unsaturated lipid egg yolk lecithin (PC-98T) and then induced the structure collapse of liposome, which was responsible for the photo-activatable release of **triptolide**. Second, ROS also executed PDT to cooperate with chemotherapy. Third, the formulation increased the water solubility of **triptolide**, and avoided leakage in the delivery, and enhanced the tumor accumulation by EPR effect and laser focusing. Therefore, the system TP/Ce6-LP greatly improved the antitumor efficacy and notably reduced its system toxicity.

#### Chemotherapy with Photothermal Therapy (PTT) and Hyperthermia Therapy

By converting light energy to heat energy, PTT increases the target’s temperature to induce cell death, with non-invasion and non-resistance. This therapy relies on the distributions, conversion efficacy, and absorbed wavelength of the photothermal agent. However, hyperthermia therapy replaces the photothermal agent and light irradiation with an external heating instrument. Thermosensitive liposomal system was often fabricated as a drug-release platform by incorporation of special lipid with a suitable phase transition temperature (T_c_) above the physiological temperature 37°C.

**FIGURE 12:**
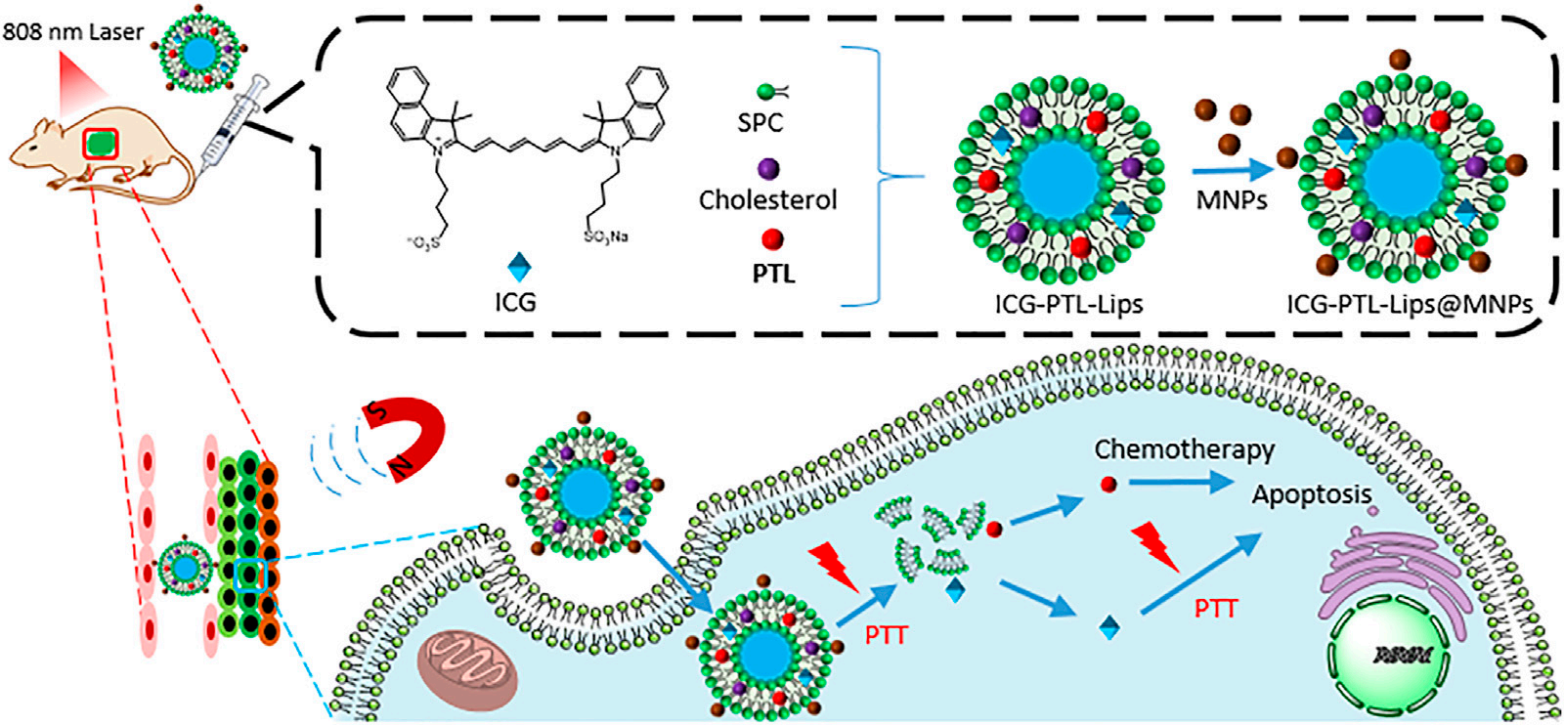
The preparation of ICG-PTL-Lips@MNPs and the mechanism of combined therapies: chemotherapy with PTT.

Nanomagnetic liposomal system ICG-PTL-Lips@MNPs encapsulated **parthenolide** as a chemotherapeutic agent, indocyanine green (ICG) as a photothermal agent and magnetic Fe_3_O_4_ nanoparticles for the targeted and chemo-PTT (Figure 12) (Gao et al., 2020a). First, the magnetic liposomes were directed to tumor sites by an external magnetic field. Second, laser irradiation (808 nm) promoted the system to release the cargoes by photothermal effect. Third, the synergistic combination of chemotherapy and PTT enhanced the antitumor efficacy and reduced the side effects.

**FIGURE 13:**
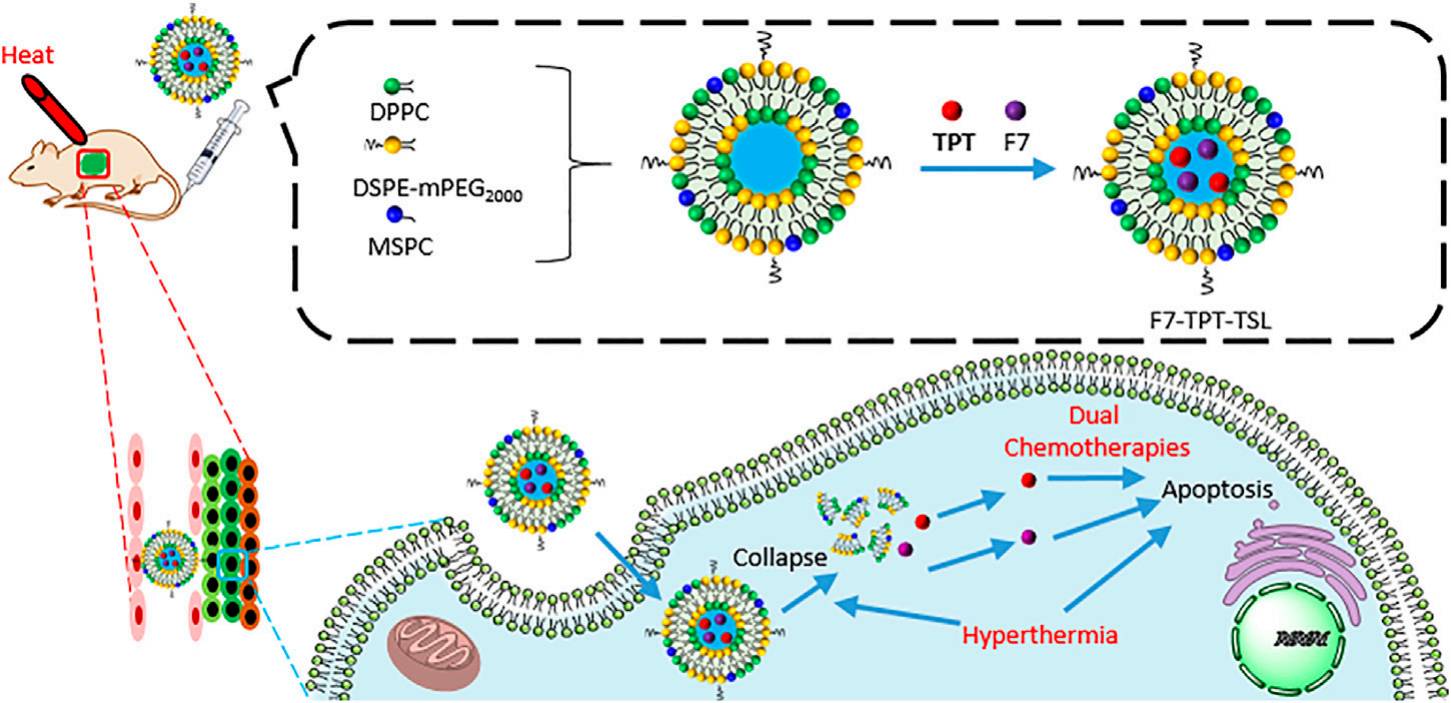
The preparation of F7-TPT-TSL and the mechanism of combined therapies: dual chemotherapies with hyperthermia therapy.

Thermosensitive liposomes (F7-TPT-TSL) co-loading two therapeutic agents **topotecan** (TPT) and F7 were fabricated with DPPC (T_c_ 41°C) and other lipids for the combined therapies (Figure 13) (Du et al., 2020). After administration of the liposomes and delivery to tumors, the tumor was heated for 30 min with a 42.5°C copper column to induce the drug release. The system significantly improved the antitumor effects and reduced systemic toxicity.

#### Chemotherapy with Radiotherapy

As one of the traditional antitumor therapies in the clinic, radiotherapy adopts a high dose of radiation to damage the DNA of cancer cells, leading to killing or shrinking tumors. However, it is hard to treat metastatic foci which is normally too tiny to be detected. The damage to surrounding normal tissue is unavoidable.

In the combined therapies, liposomal **honokiol** as a chemotherapeutic agent sensitized the tumor cells to radiotherapy (Hu et al., 2008). The combined therapies delayed the tumor growth (8.7 days) and significantly improved the survival time, compared with single treatments.

#### Chemotherapy with Chemodynamic Therapy (CDT) and Starvation Therapy

**FIGURE 14:**
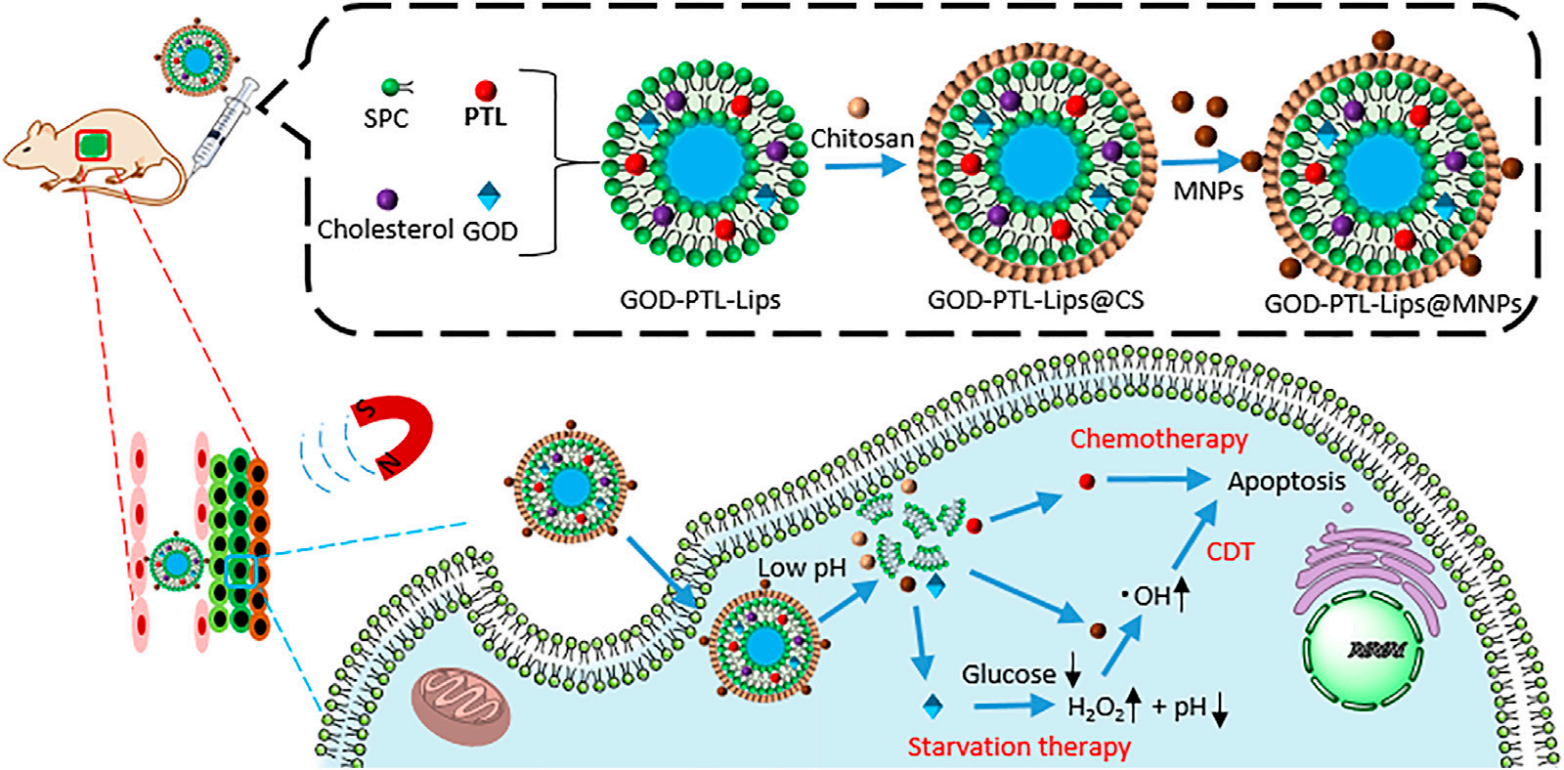
The preparation of GOD-PTL-Lips@MNPs and the mechanism of combined therapies: chemotherapy with starvation and CDT.

Multifunctional liposomes GOD-PTL-Lips@NMPs encapsulating **parthenolide** and glucose oxidase, were modified with Fe_3_O_4_ magnetic nanoparticles and **chitosan** (a linear polysaccharide) for combined therapies (Figure 14) (Gao et al., 2020b). First, the nanoparticles were delivered to tumor tissue by EPR effects and magnetic targeting. Second, after cellular uptake, due to the protonation of -NH_2_ group, chitosan induced the liposomes to collapse and release the cargoes. Third, Fe_3_O_4_ magnetic nanoparticles not only guided the delivery by the magnetic field, but also catalyzed the Fenton reaction for CDT. Fourthly, glucose oxidase (GOD) consumed glucose to starve the targeted cells and generated H_2_O_2_ to enhance CDT. Fifthly, **parthenolide** as a chemotherapeutic agent not only induced cell apoptosis, but also consumed GSH to enhance CDT.

### Image-Guided Therapy

Personalized medicine required that the treatment was performed according to the personalities of the patients. By co-delivery of therapeutic and imaging contrast agents, multifunctional liposome as a theranostic platform allowed various diagnostic tests to guide therapy and evaluate the therapeutic outcome. As a review recently summarized, liposome-based imaging approaches have visualized the fate of liposomes *in vivo*, such as computed tomography (CT), magnetic resonance imaging (MRI), positron emission tomography (PET) imaging, photoacoustic imaging (PAI), and FLI (Xia et al., 2019).

**FIGURE 15:**
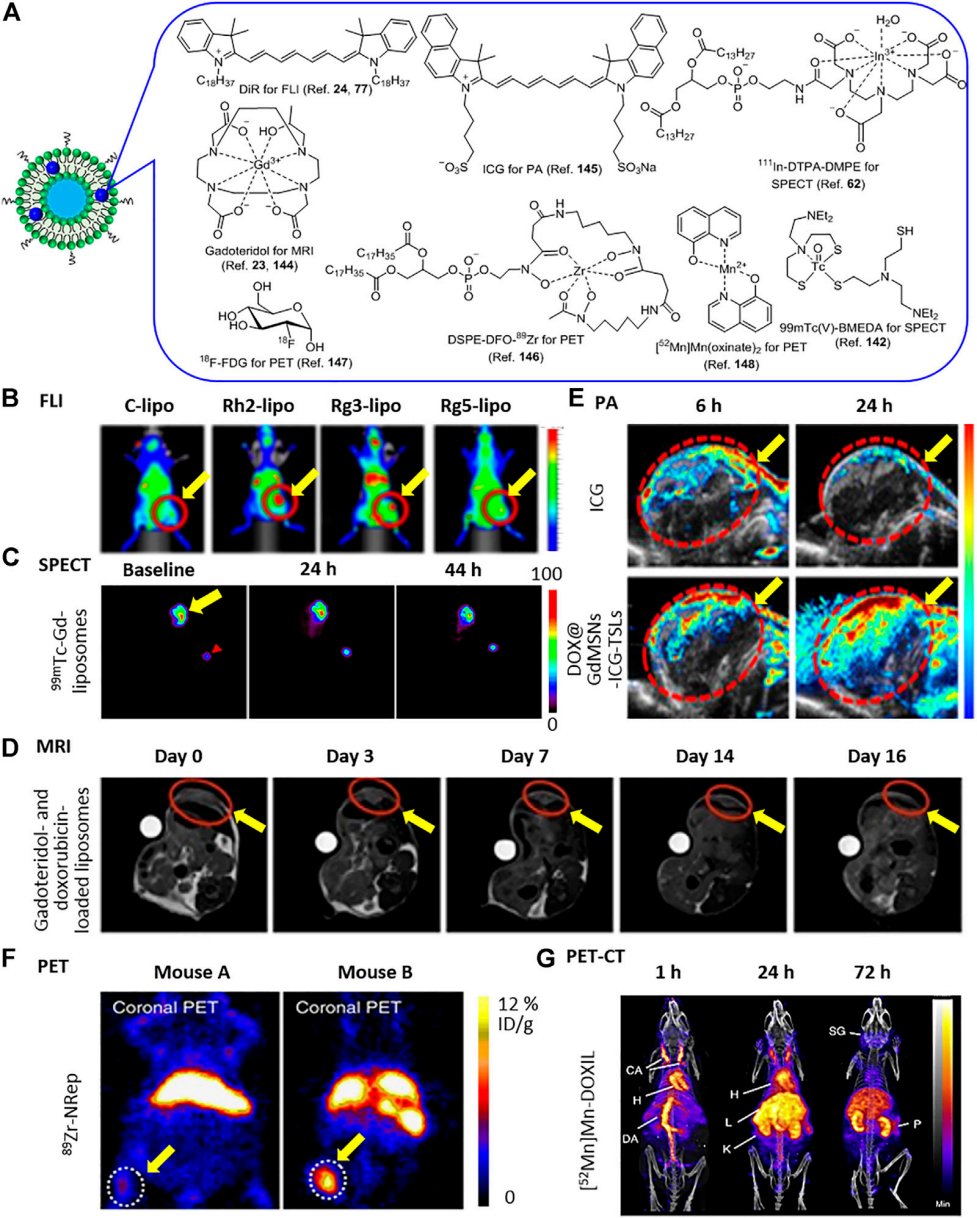
**(A)** Imaging contrast agents. **(B)** FLI of BGC-823 tumor-bearing mice after i.v. injection of DiR-labeled C-lipo and ginsenoside liposomes (C-lipo, Rh2-lipo, Rg3-lipo, and Rg5-lipo). Reproduced (Adapted) under the terms of the Creative Commons Attribution (CC BY-NC 4.0) (Hong et al., 2019). Copyright 2019, Theranostics. **(C)** SPECT imaging of SCCHN tumor-bearing nude rats after i.v. injection of ^99m^Tc-Gd-liposomes. Reprinted (adapted) with permission (Li et al., 2012b). Copyright 2012, American Chemical Society. **(D)** MR images of after i.v. injection of sonosensitive gadoteridol- and doxorubicin-loaded liposomes. Reproduced (Adapted) under Creative Commons Attribution License (CC BY) (Shi et al., 2015). Copyright 2020, Patrucco and Terreno; **(E)** PA images after i.v. injection of free ICG and DOX@GdMSNs-ICG-TSLs. Reprinted (adapted) with permission (Sun et al., 2018). Copyright 2018, American Chemical Society. **(F)** PET Images of 4T1 tumor-bearing mice with low ^89^Zr-NRep uptake (mouse A, left) and high ^89^Zr-NRep uptake (mouse B, right) after i.v. injection. Reproduced (Adapted) under Creative Commons Attribution License (CC BY) (Pérez-Medina et al., 2016). Copyright 2016, Mulder and Reiner et al. **(G)**, PET-CT imaging of a B6CBAF1 mouse after i.v. injection of [^52^Mn]Mn-DOXIL. Reproduced (Adapted) with permission (Gawne et al., 2018). Copyright 2018, Royal Society of Chemistry. The yellow arrow indicated tumors. Red arrowhead indicated the outside reference standard.

FLI has high sensitivity and resolution. Long wavelengths of dye’s absorption and emission, such NIR region, could significantly improve tissue penetration. Before encapsulated in liposomes, a fluorescent probe derived from **paclitaxel** (PTX) by chemically conjugation to a NIR fluorescent dye DiR (Figure 15A) (Shi et al., 2015). DiR-labeled liposomes modified with **ginsenosides**, clearly visualized their *in vivo* distributions at different time points in BGC-823 tumor-bearing mice (Figure 15A) (Hong et al., 2019). The images showed that **ginsenosides** had guided the liposomes encapsulating **paclitaxel** (PTX) to tumor site, compared C-lipo group with Rh2-, Rg3-, and Rg5-lipo groups (Figures 15A,B).

Single-photon emission computed tomography (SPECT) reconstructs the 3D information of the target by detecting gamma rays from radionuclide. ^111^In-labeled liposomes encapsulating ^111^In and **CPT** were delivered to an HT-29 tumor-bearing mouse (Figure 15A) (Flaten et al., 2013). The combination of SPECT images showed that the liposomes mainly accumulated in the liver at 2 h postinjection and the intestine slowly developed to be the second-highest site during 20 h. In another case, SPECT image showed a high accumulation of ^99m^Tc-Gd-liposomes in the tumor at 44 h post-injection (Figures 15A,C) (Li et al., 2012b).

MRI is a popular technique for medical checking imaging in the clinic by detecting protons in water and fat molecules in the body. To improve the sensitivity, contrast agents are often administrated. A temperature-sensitive liposome-encapsulated **doxorubicin** and gadoteridol [Gd(HPDO3A)(H_2_O)] as a paramagnetic T1 contrast agent of MRI (Figure 15A) (de Smet et al., 2010). MRI also monitored the release process of **doxorubicin** and gadoteridol in tumor from thermal- and sono-sensitive liposomes, and the therapeutic outcome (Figures 15A,D) (Patrucco and Terreno, 2020). In an earlier study, MRI was applied to the therapy by liposomes encapsulating **doxorubicin** and an old contrast agent MnSO_4_ (Viglianti et al., 2004). In another case, Fe_3_O_4_ nanoparticles served as T2 contrast agents of MRI and were encapsulated in liposomes to visualize the combined therapies of **parthenolide** and other therapeutic agents (Gao et al., 2020b).

PA imaging is a non-toxic, non-invasive technique with excellent penetration and high spatial resolution, by converting light into heat and acoustic signal. PA imaging visualized the bio-distributions of liposomes encapsulating ICG as photothermal agent and **doxorubicin**, and the synergistic therapeutic effects of chemotherapy and PDT (Figures 15A,E) (Sun et al., 2018).

Currently, PET is the only functional imaging technique to afford metabolic information which is extremely suitable for tumor and metastases diagnosis, while others only offer anatomic information. It relies on the tracer containing the positron-emitting radioisotopes, such as ^18^F, ^52^Mn, ^64^Cu, ^89^Zr and so on. ^89^Zr-labeled liposomes facilitated the quantification of the **doxorubicin** accumulation in tumors (Figures 15A,F) (Pérez-Medina et al., 2016).

Compared with single imaging, dual-modal imaging has extra advantages. For example, due to the limitation in only metabolic information, PET is normally combined with MRI and CT for anatomic information in clinic. PET-MRI clearly visualized the location, morphology, and activity of the tumor before and after treatment with liposomal **doxorubicin** (Figure 15A) (Zimmermann et al., 2017). ^52^Mn-labeled Doxil^®^ revealed the long circulation and distributions in organs by PET-CT (Figures 15A,G) (Gawne et al., 2018) as well as ^64^Cu-liposome (Lee et al., 2018). Similarly, SPECT-CT also offered the information on distributions of liposomal **doxorubicin** and liposomal **vinorelbine**, and validated the absence of competing effect (Wu et al., 2017). ^188^Re-labeled nanoliposomes visualized the treatment of **doxorubicin** in human colorectal adenocarcinoma-bearing mice by SPECT-CT (Chen et al., 2010).

## Conclusion and Prospective

The tunable size, biocompatible nature, triggerable cargo release, surface modifications for targeted delivery, and physical compartment make the functional liposome as an ideal carrier for natural products and their analogs with higher efficiency and less toxicity.

More and more natural products are isolated and identified with abundant biological activities, due to biological evolutions for survival advantage. Many of them have the potential to be therapeutic agents, which can be encapsulated into multifunctional liposomes for better therapeutic effects.

TCM has many positive results in therapy, although the mechanism of most TCM still remained unclear, such as upregulation of tumor-related T cells (Hoffman et al., 2020). To reduce unexpected side effects, liposomal formulation is a good option.

Liposome not only delivers nature products but also biomacromolecules, such as DNA (Guo et al., 2018), RNA (Hou et al., 2021), protein (Swaminathan and Ehrhardt, 2012), and bacteriophages (Nieth et al., 2015) for therapies. Therefore, it is the potential to improve the therapeutic effects by combined therapy in cooperation with various drugs based on different mechanisms.

Overall, the liposomal system still suffered limited efficiency in targeted delivery to the lesion. For example, developing new techniques to help the liposomal delivery to overcome biological barriers, such as blood-brain barrier and blood-tumor barrier, are also challenges in drug delivery. On one hand, new materials such as cancer membrane, synthetic lipid, blood cell membrane, have exhibited promising advantages in better biocompatibility and targeted delivery. Furthermore, engineering strategy was applied to construct an intelligent platform, such as a liposome-based robot (Inaba et al., 2018; Shoji and Kawano, 2020), bacterial motors (Zhang et al., 2013b; Dogra et al., 2016) and so on. On the other hand, physical tool, such as focused ultrasound, has improved the delivery efficiency of liposomal **paclitaxel** to mice brain and tumor (Shen et al., 2017).

Although liposome-based molecular imaging has experienced explosive development (Xia et al., 2019), such as FLI, MRI, CT, PET, ultrasound imaging and PA imaging, developing new imaging agents suitable for liposome-labeling, will further promote medicinal nature products-involved image-guided therapy.

In general, liposomal formulation has experienced fast developments in targeted therapy, biocompatibility, safety, and so on. Undoubtedly, as a carrier of medicinal natural products and their derivatives, it plays a very important role in the drug formulation and will significantly promote drug discovery.
